# Susceptibility of ABO blood group to COVID-19 infections: clinico-hematological, radiological, and complications analysis

**DOI:** 10.1097/MD.0000000000028334

**Published:** 2021-12-30

**Authors:** Saeed M. Kabrah, Samer S. Abuzerr, Mohammed A. Baghdadi, Ahmed M. Kabrah, Arwa F. Flemban, Fayez S. Bahwerth, Hamza M. Assaggaf, Eisa A. Alanazi, Abdullah A. Alhifany, Sarah A. Al-Shareef, Wesam H. Alsabban, Anmar A. Khan, Hissah Alzhrani, Layal K. Jambi, Radi T. Alsafi, Akhmed Aslam, Hebah M. Kabrah, Ahmad O. Babalghith, Amr J. Halawani

**Affiliations:** a Laboratory Medicine Department, Faculty of Applied Medical Sciences, Umm Al-Qura University, Makkah, Kingdom of Saudi Arabia; b Visiting Scholar with the School of Public Health, Department of Social and Preventive Medicine, University of Montreal, Montreal, Canada; c Quality Improvement and Infection Control Unit, Ministry of Health, Gaza, Palestine; d Research Centre, King Faisal Specialist Hospital and Research Centre Jeddah, Kingdom of Saudi Arabia; e Pathology Department, Faculty of Medicine, Umm Al-Qura University, Makkah, Kingdom of Saudi Arabia; f Laboratory and Blood Bank Department, King Faisal Hospital, Makkah, Kingdom of Saudi Arabia; g Computer Science Department, Faculty of Computer and Information System, Umm Al-Qura University, Makkah, Kingdom of Saudi Arabia; h Clinical Pharmacy Department, College of Pharmacy, Umm Al-Qura University, Makkah, Kingdom of Saudi Arabia; i Information System Department, Faculty of Computer and Information Systems, Umm Al Qura University, Makkah, Kingdom of Saudi Arabia; j Mathematical Sciences Department, Faculty of Applied Sciences, Umm Al-Qura University, Makkah, Kingdom of Saudi Arabia; k Radiological Sciences Department, College of Applied Medical Sciences, King Saud University, Riyadh, Kingdom of Saudi Arabia; l Laboratory and Blood Bank Department, Al Noor Specialist Hospital, Makkah, Kingdom of Saudi Arabia; m Medical Genetic Department, Faculty of Medicine, Umm Al-Qura University, Makkah, Kingdom of Saudi Arabia.

**Keywords:** ABO blood group, COVID-19, infections, protocols, susceptibility, symptoms

## Abstract

In the wake of the COVID-19 pandemic, research indicates that the COVID-19 disease susceptibility varies among individuals depending on their ABO blood groups. Researchers globally commenced investigating potential methods to stratify cases according to prognosis depending on several clinical parameters. Since there is evidence of a link between ABO blood groups and disease susceptibility, it could be argued that there is a link between blood groups and disease manifestation and progression. The current study investigates whether clinical manifestation, laboratory, and imaging findings vary among ABO blood groups of hospitalized confirmed COVID-19 patients.

This retrospective cohort study was conducted between March 1, 2020 and March 31, 2021 in King Faisal Specialist Hospital and Research Centre Riyadh and Jeddah, Saudi Arabia. Demographic information, clinical information, laboratory findings, and imaging investigations were extracted from the data warehouse for all confirmed COVID-19 patients.

A total of 285 admitted patients were included in the study. Of these, 81 (28.4%) were blood group A, 43 (15.1%) were blood group B, 11 (3.9%) were blood group AB, and 150 (52.6%) were blood group O. This was almost consistent with the distribution of blood groups among the Saudi Arabia community. The majority of the study participants (79.6% [n = 227]) were asymptomatic. The upper respiratory tract infection (*P* = .014) and shortness of breath showed statistically significant differences between the ABO blood group (*P* = .009). Moreover, the incidence of the symptoms was highly observed in blood group O followed by A then B except for pharyngeal exudate observed in blood group A. The one-way ANOVA test indicated that among the studied hematological parameters, glucose (*P* = .004), absolute lymphocyte count (*P* = .001), and IgA (*P* = .036) showed statistically significant differences between the means of the ABO blood group. The differences in both X-ray and computed tomography scan findings were statistically nonsignificant among the ABO age group. Only 86 (30.3%) patients were admitted to an intensive care unit, and the majority of them were blood groups O 28.7% (n = 43) and A 37.0% (n = 30). However, the differences in complications’ outcomes were statistically nonsignificant among the ABO age group.

ABO blood groups among hospitalized COVID-19 patients are not associated with clinical, hematological, radiological, and complications abnormality.

## 1 Introduction

In December 2019, the new coronavirus illness COVID-19, caused by the severe acute respiratory syndrome coronavirus-2, was discovered in Wuhan, China. COVID-19 infection quickly became a worldwide pandemic, impacting nearly all nations and posing a public health threat.^[1]^ There is much evidence that blood antigens have a role in COVID-19 etiology.^[2]^

Blood group antigens are known to affect the innate immune system's responses and allow pathogen absorption and signal transduction.[^3^,^4^,^5^] For instance, the risk of hepatitis B virus infection was considerably lower in those with blood type O, according to Mohammadali et al.^[2]^ Elnady et al observed that rotavirus gastroenteritis was more common in blood type A people and considerably less common in blood type B people.^[6]^ Another research by Degarege et al found that those with blood group A, malaria had a greater risk of anemia than people with non-A phenotypic.^[7]^ Murugananthan et al discovered that individuals with the AB blood group had a 2.5-fold increased chance of having dengue hemorrhagic fever than those with any other blood type.^[8]^

Due to specific underlying biological characteristics, blood type O has been linked to a decreased risk of diseases such as diabetes, atherosclerosis, heart disease, and some infections.[^9^,^10^,^11^,^12^] It is debatable if these associations lead to clinically worse results in various blood types. A systematic review meta-analysis has recently shown that the COVID-19 infection rate was more likely to be observed in persons with blood group A > O > B > AB; also, the study indicated substantial variation between observation research that showed a correlation between ABO blood group and COVID-19.^[13]^. Nevertheless, no significant differences were found in several other observational investigations.[^14^,^15^,^16^]

Because severe acute respiratory syndrome coronavirus-2 is a novel virus, it is still unknown if ABO blood groups influence an individual's susceptibility or severity of illness. Accordingly, the current study aims at investigating whether clinical manifestation, laboratory evaluation, and imaging findings vary among ABO blood groups of hospitalized confirmed COVID-19 patients; this will add to the growing body of evidence suggesting blood group may play a role in COVID-19.

## 2 Materials and methods

### 2.1 Study design and study population

The current research is a retrospective cohort analysis of hospitalized confirmed COVID-19 patients in King Faisal Specialist Hospital and Research Centre (KFSH&RC) Riyadh and Jeddah, Saudi Arabia. It included all confirmed COVID-19 patients with complete investigation admitted to the hospital between the March 1, 2020 and the March 31, 2021.

### 2.2 Data collection

Medical records were obtained from the data warehouse for all confirmed COVID-19 patients diagnosed according to the WHO guideline with a positive result for nasal and pharyngeal swab specimens analyzed by real-time reverse transcriptase-polymerase chain reaction assay. Extracted data included demographic information, clinical information, laboratory findings, and imaging investigations. All radiological images were analyzed and diagnosed by three radiologists, each with 3 to 4 years of experience in reporting various chest X-ray and computed tomography (CT) examinations. All hematological and radiologic examinations were conducted at day fourth of infection.

### 2.3 Ethical approval

The study was approved by the KFSH&RC institute review board (RC-J/550/41) and the biomedical ethics committee faculty of medicine at Umm Al-Qura University (HAPO-02-K-012-2020-06-393).

### 2.4 Statistical analysis

The data analysis was made using the Statistical Packages for Social Sciences (SPSS) version 24 (IBM Corp, Armonk, NY). Descriptive statistics of frequency and percentage and mean and standard deviation were performed for categorical and continuous variables, respectively. The chi-square test was used to examine differences in the prevalence of different categorical variables. The one-way ANOVA test was applied to investigate the differences between the ABO blood group means. A *P*-value < .05 was considered statistically significant.

## 3 Results

A total of 60,000 patients were admitted to the KFSH&RC and diagnosed with COVID-19 infection between the March 1, 2020 and the March 31, 2021. Altogether 285 patients were included in this study due to the missing clinical information and/or laboratory and imaging investigation. Of these cases, 81 (28.4%) were blood group A, 43 (15.1%) were blood group B, 11 (3.9%) were blood group AB, and 150 (52.6%) were blood group O. Majority of patients (71.2%) were of age group 25–64 years and 104 (36.5%) were of blood group O. Also, 50.9% (n = 145) participants were females, and 49.1% (n = 140) were males. The nationality of the study participants showed statistically significant differences between the ABO blood group at *P* < .05 as most participants were Saudi (81.1%). Based on body mass index, 38.2% (n = 109) and 33.7% (n = 96) of the study participants were of overweight and obese status, respectively. Only 3.5% (n = 10) of the study participants were pregnant women. Of the study participants, only 8.8% (n = 25) were smokers, and the smoking status of the study participants showed statistically significant differences between the ABO blood group at *P* < .05. Most of the smokers, 7.0% (n = 20), belong to blood group O (Table 1).

**Table 1:** Demographic and clinical features according to ABO blood group.

Variables	Categories	A	B	AB	O	*X* ^2^	*P*
Total (%)	285 (100)	81 (28.4)	43 (15.1)	11 (3.9)	150 (52.6)		
Age group	<14	0 (0.0)	0 (0.0)	0 (0.0)	2 (1.3)	5.225	.814
	15–24	6 (7.4)	5 (11.6)	0 (0.0)	16 (10.7)		
	25–64	62 (76.5)	28 (65.1)	9 (81.8)	104 (69.3)		
	>65	13 (16.0)	10 (23.3)	2 (18.2)	28 (18.7)		
Gender	Male	32 (39.5)	22 (51.2)	6 (54.5)	80 (53.3)	4.262	.234
	Female	49 (60.5)	21 (48.8)	5 (45.5)	70 (46.7)		
Nationality	Saudi	64 (79.0)	27 (62.8)	8 (72.7)	132 (88)	14.768	.002
	Non-Saudi	17 (21.0)	16 (37.2)	3 (27.3)	18 (12)		
Body mass index	<18.5	2 (2.5)	0 (0)	0 (0)	8 (5.3)	8.188	.515
	18.5–24.9	21 (25.9)	13 (30.2)	3 (27.3)	33 (22.0)		
	25.0–29.9	29 (35.8)	13 (30.2)	3 (27.3)	64 (42.7)		
	>30.0	29 (35.8)	17 (39.5)	5 (45.6)	45 (30.0)		
Pregnant	Yes	3 (3.7)	4 (9.3)	0 (0.0)	3 (2.0)	9.965	.126
	No	46 (56.8)	17 (39.5)	5 (45.5)	67 (44.7)		
Smoker	Yes	3 (3.7)	2 (4.7)	0 (0.0)	20 (13.3)	8.470	.037
	No	78 (96.3)	41 (95.3)	11 (100.0)	130 (86.7)		

There was a statistically significant difference in the incidence of upper respiratory tract infection (*P* = .014) and shortness of breath among participants with different ABO blood groups (*P* = .009). About 79.6% (n = 227) of the study participants were asymptomatic (as seen in Table 2), and the remaining 20.4% (n = 58) showed varying degrees of symptoms. The clinical symptoms were more likely to be observed in participants with O, A, and B blood groups, respectively, except for pharyngeal exudate that was more likely to be observed in cases with blood group A. Haemoptysis, jaundice, abnormal auscultation of the lung and hepatomegaly were reported only in patients with O blood groups.

**Table 2:** Symptoms variation between ABO blood groups.

Variables	Categories	A	B	AB	O	*X* ^2^	*P*	Incidence
Total	285 (100)	81 (28.4)	43 (15.1)	11 (3.9)	150 (52.6)			O>A>B>AB
Asymptomatic	Yes	16 (19.8)	11 (25.6)	2 (18.2)	29 (19.3)	0.871	.832	O>A>B>AB
	No	65 (80.2)	32 (74.4)	9 (81.8)	121 (80.7)			O>A>B>AB
Fever (≥38°C)	Yes	27 (33.3)	16 (37.2)	3 (27.3)	55 (36.7)	0.635	.888	O>A>B>AB
	No	54 (66.7)	27 (62.8)	8 (72.7)	95 (63.3)			O>A>B>AB
Feverish/chills	Yes	23 (28.4)	7 (16.3)	2 (18.2)	44 (29.3)	5.076	.534	O>A>b>AB
	No	58 (71.6)	36 (83.7)	9 (81.8)	106 (70.7)			O>A>B>AB
Sore throat	Yes	21 (25.9)	9 (20.9)	5 (45. 5)	52 (34.7)	5.044	.169	O>A>B>AB
	No	60 (74.1)	34 (79.1)	6 (54.5)	98 (65.3)			O>A>B>AB
Runny nose	Yes	13 (16.0)	4 (9.3)	1 (9.1)	26 (17.3)	2.015	.569	O>A>B>AB
	No	68 (84.0)	39 (90.7)	10 (90.9)	124 (82.7)			O>A>B>AB
Myalgia fatigue	Yes	22 (27.2)	8 (18.6)	1 (9.1)	47 (31.3)	4.698	.195	O>A>B>AB
	No	59 (72.8)	35 (81.4)	10 (90.9)	103 (68.7)			O>A>B>AB
Anorexia	Yes	4 (4.9)	4 (9.3)	0 (0)	13 (8.7)	2.182	.536	O>A=B
	No	77 (95.1)	39 (91.0)	11 (100.0)	137 (91.3)			O>A>B>AB
URTI	Yes	20 (24.7)	2 (4.7)	0 (0.0)	24 (16.0)	10.689	.014	O>A>B>AB
	No	61 (75.3)	41 (95.3)	11 (100.0)	126 (84.0)			O>A>B>AB
Dry cough	Yes	35 (43.2)	13 (30.2)	3 (27.3)	49 (32.7)	3.472	.324	O>A>B>AB
	No	46 (56.8)	30 (69.8)	8 (72.7)	101 (67.3)			O>A>B>AB
Productive cough	Yes	15 (18.5)	4 (9.3)	1 (9.0)	26 (17.3)	2.386	.496	O>A>B>AB
	No	66 (81.5)	39 (90.7)	10 (90.9)	124 (82.7)			O>A>B>AB
Shortness of breath	Yes	21 (25.9)	5 (11.6)	2 (18.1)	36 (24.0)	17.068	.009	O>A>B>AB
	No	60 (74.1)	38 (88.4)	9 (81.8)	114 (76.0)			O>A>B>AB
Chest pain	Yes	8 (9.9)	2 (4.7)	1 (9.1)	8 (5.3)	2.154	.541	O=A>B>AB
	No	73 (90.1)	41 (95.3)	10 (91.0)	142 (94.7)			O>A>B>AB
Hemoptysis	Yes	0 (0.0)	0 (0.0)	0 (0.0)	2 (1.3)	1.813	.612	O
	No	81 (100.0)	43 (100.0)	11 (100.0)	148 (98.7)			O>A>B>AB
Headache	Yes	21 (25.9)	6 (14.0)	2 (18.2)	33 (22.0)	3.376	.760	O>A>B>AB
	No	60 (74.1)	37 (86.0)	9 (81.8)	117 (78.0)			O>A>B>AB
Confusion	Yes	1 (1.2)	3 (7.0)	0 (0.0)	3 (2.0)	4.580	.205	O>B>A
	No	80 (98.8)	40 (93.0)	11 (100.0)	147 (98.0)			O>A>B>AB
Loss of consciousness	Yes	3 (3.7)	1 (2.3)	0 (0.0)	2 (1.3)	1.685	.640	O>A>B
	No	78 (96.3)	42 (97.6)	11 (100.0)	148 (98.7)			O>A>B>AB
Seizure	Yes	1 (1.2)	0 (0.0)	0 (0.0)	1 (0.7)	0.714	.870	O=A
	No	80 (98.8)	43 (100.0)	11 (100.0)	149 (99.3)			O>A>B>AB
Nausea	Yes	7 (8.6)	5 (11.6	0 (0.0)	17 (11.3	1.774	.621	O>A>B
	No	74 (91.4)	38 (88.4)	11 (100.0)	133 (88.7)			O>A>B>AB
Vomiting	Yes	7 (8.6)	7 (11.6)	0 (0.0)	12 (8.0)	4.011	.260	O>A=B
	No	74 (91.4)	36 (88.4)	11 (100.0)	138 (92.0)			O>A>B>AB
Abdominal pain	Yes	5 (6.2)	5 (16.3)	0 (0.0)	8 (5.3)	3.040	.386	O>A=B
	No	76 (93.8)	38 (83.7)	11 (100.0)	142 (94.7)			O>A>B>AB
Diarrheal	Yes	15 (18.5)	10 (23.2)	1 (9.1)	25 (16.7)	1.597	.660	O>A>B>AB
	No	66 (81.5)	33 (76.7)	10 (90.0)	125 (83.3)			O>A>B>AB
Jaundice	Yes	0 (0.0)	0 (0.0)	0 (0.0)	1 (0.7	0.903	.825	O
	No	81 (100.0)	43 (100.0)	11 (100.0)	149 (99.3			O>A>B>AB
Altered mental status	Yes	2 (2.5)	2 (4.6)	1 (9.1)	8 (5.3)	1.539	.673	O>A=B>AB
	No	79 (97.5)	41 (95.3)	10 (90.0)	142 (94.7)			O>A>B>AB
Conjunctival injection (eye redness)	Yes	1 (1.2)	0 (0.0)	0 (0.0)	1 (0.7)	0.714	.870	O=A
	No	80 (98.8)	43 (100.0)	11 (100.0)	149 (99.3)			O>A>B>AB
Pharyngeal exudate	Yes	1 (1.2)	0 (0.0)	0 (0.0)	0 (0.0)	2.527	.470	A
	No	80 (98.8)	43 (100.0)	11 (100.0)	150 (100.0)			O>A>B>AB
Abnormal auscultation of the lung	Yes	0 (0.0)	0 (0.0)	0 (0.0)	1 (0.7)	0.903	.825	O
	No	81 (100.0)	43 (100.0)	11 (100.0)	149 (99.3)			O>A>B>AB
Hypopigmentation (skin examination)	Yes	0 (0.0)	0 (0.0)	0 (0.0)	0 (0.0)	-	-	-
	No	81 (100.0)	43 (100.0)	11 (100.0)	150 (100.0)			O>A>B>AB
Rash	Yes	1 (1.2)	0 (0.0)	0 (0.0)	5 (3.3)	2.557	.465	O>A
	No	80 (98.9)	43 (100.0)	11 (100.0)	145 (96.7)			O>A>B>AB
Splenomegaly	Yes	0 (0.0)	0 (0.0)	0 (0.0)	0 (0.0)	-	-	-
	No	81 (100.0)	43 (100.0)	11 (100.0)	150 (100.0)			O>A>B>AB
Hepatomegaly	Yes	0 (0.0)	0 (0.0)	0 (0.0)	1 (0.7)	0.903	.825	O
	No	81 (100.0)	43 (100.0)	11 (100.0)	149 (99.3)			O>A>B>AB
ECG rhythm	Regular	71 (87.7)	38 (88.4)	10 (91.0)	134 (89.3)	0.206	.977	O>A>B>AB
	Irregular	10 (12.3)	5 (11.6)	1 (9.1)	16 (10.7)			O>A>B>AB
Echocardiography	Normal	75 (92.6)	39 (90.7)	10 (90.9)	140 (93.3)	0.392	.942	O>A>B>AB
	Abnormal	6 (7.4)	4 (9.3)	1 (9.1)	10 (6.7)			O>A>B>AB

There was some observational difference in the hematological parameters among participants with different ABO blood groups (as seen in Table 3). One-way ANOVA test indicated that among the studied hematological parameters, glucose (*P* = .004), absolute lymphocyte count (*P* = .001), and IgA (*P* = .036) showed statistically significant differences between the means of cases with different ABO blood groups (Table 3). The mean values of several hematological parameters exceeded the normal levels in cases, including that of prothrombin time (15.8 seconds), partial thromboplastin time (39.0 seconds), partial pressure of carbon dioxide (PCO_2_, 6.5 kPa), lactate (1.6 mmol/L), ferritin (444.9 ng/mL), CD4 (534.6 cells/mm^3^), fibrinogen (4.4 g/L), haptoglobin (2.5 g/L), erythrocyte sedimentation rate (56.1 mm/hour), beta 2 microglobulin (3.1 μg/mL), and soluble transferrin receptor (5.7 mg/L). The mean values of several other hematological parameters were, on the other hand, lower than normal levels in cases including the platelet (208.7/μL if blood), urea (6.3 mg/dL), bicarbonate (21.9 mmol/L), glucose (7.4 mmol/L), magnesium (0.8 mmol/L), and iron (8.5 μmol/L).

**Table 3:** Hematological parameters variation between ABO blood groups.

		Sum of squares	df	Mean square	Mean ± SD	*F*	*P*
Hemoglobin (g/dL)	Between groups	723.642	3	241.214	15.5 ± 17.5	0.788	.501
	Within groups	86,018.593	281	306.116			
	Total	86,742.235	284				
Platelet (cells/μL)	Between groups	11,814.062	3	3938.021	208.7 ± 71.5	0.768	.513
	Within groups	1,440,263.924	281	5125.494			
	Total	1,452,077.986	284				
Hematocrit (%)	Between groups	0.009	3	0.003	0.4 ± 0.1	0.856	.464
	Within groups	1.018	281	0.004			
	Total	1.027	284				
Prothrombin time (PT) (s)	Between groups	68.690	3	22.897	15.8 ± 5.0	0.897	.443
	Within groups	7169.537	281	25.514			
	Total	7238.227	284				
Partial thromboplastin time (PTT) (s)	Between groups	34.677	3	11.559	39.0 ± 6.8	0.246	.864
	Within groups	13,180.743	281	46.907			
	Total	13,215.420	284				
INR	Between groups	3.995	3	1.332	1.3 ± 2.5	0.215	.886
	Within groups	1741.171	281	6.196			
	Total	1745.166	284				
Urea (mmol/L)	Between groups	91.775	3	30.592	6.3 ± 7.1	0.599	.616
	Within groups	14,342.975	281	51.043			
	Total	14,434.749	284				
Creatinine (mmol/L)	Between groups	5012.062	3	1670.687	87.3 ± 78.9	0.266	.850
	Within groups	1,762,197.721	281	6271.166			
	Total	1,767,209.783	284				
Sodium (mEq/L)	Between groups	10.998	3	3.666	138.6 ± 5.2	0.137	.938
	Within groups	7521.971	281	26.769			
	Total	7532.968	284				
Potassium (mmol/L)	Between groups	0.299	3	0.100	4.2 ± 0.6	0.266	.850
	Within groups	105.461	281	0.375			
	Total	105.760	284				
Bicarbonate (mmol/L)	Between groups	36.083	3	12.028	21.9 ± 3.6	0.905	.439
	Within groups	3732.842	281	13.284			
	Total	3768.925	284				
Glucose (mmol/L)	Between groups	181.073	3	60.358	7.4 ± 3.7	4.562	.004
	Within groups	3717.645	281	13.230			
	Total	3898.719	284				
Magnesium (mmol/L)	Between groups	0.060	3	0.020	0.8 ± 0.1	1.270	.285
	Within groups	4.404	281	0.016			
	Total	4.464	284				
Calcium (mmol/L)	Between groups	0.025	3	0.008	2.2 ± 0.2	0.247	.863
	Within groups	9.295	281	0.033			
	Total	9.320	284				
Phosphate (mmol/L)	Between groups	0.045	3	0.015	1.1 ± 0.3	0.164	.921
	Within groups	25.780	281	0.092			
	Total	25.825	284				
Alanine aminotransferase (ALT) (units/L)	Between groups	886.433	3	295.478	29.8 ± 21.9	0.612	.608
	Within groups	135,614.430	281	482.614			
	Total	136,500.863	284				
Aspartate aminotransferase (AST) (units/L)	Between groups	1406.072	3	468.691	33.3 ± 23.2	0.871	.456
	Within groups	151,187.372	281	538.033			
	Total	152,593.444	284				
Alkaline phosphate (international units/L)	Between groups	9375.843	3	3125.281	92.3 ± 81.7	0.465	.707
	Within groups	1,887,868.109	281	6718.392			
	Total	1,897,243.952	284				
Bilirubin (mmol/L)	Between groups	84.433	3	28.144	6.7 ± 3.9	1.890	.131
	Within groups	4184.168	281	14.890			
	Total	4268.601	284				
Creatine kinase (CK) (units/L)	Between groups	28,419.286	3	9473.095	121.9 ± 201.3	0.232	.874
	Within groups	11,479,834.760	281	40,853.504			
	Total	11,508,254.050	284				
Ph	Between groups	0.150	3	0.050	7.4 ± 0.3	0.563	.640
	Within groups	24.882	281	0.089			
	Total	25.032	284				
The partial pressure of carbon dioxide (PCO_2_) (kPa)	Between groups	20.647	3	6.882	5.8 ± 4.1	0.399	.754
	Within groups	4844.285	281	17.239			
	Total	4864.932	284				
The partial pressure of oxygen (PO_2_) (kPa)	Between groups	32.312	3	10.771	6.5 ± 4.5	0.524	.666
	Within groups	5775.427	281	20.553			
	Total	5807.739	284				
The ratio of arterial oxygen partial pressure to fractional inspired oxygen (PO_2_/FiO_2_) (mmHg)	Between groups	1400.949	3	466.983	105.8 ± 47.4	0.206	.892
	Within groups	637,956.633	281	2270.308			
	Total	639,357.583	284				
Lactate (mmol/L)	Between groups	4.239	3	1.413	1.6 ± 0.8	2.384	.070
	Within groups	166.542	281	0.593			
	Total	170.780	284				
Anion gap (mEq/L)	Between groups	47.242	3	15.747	10.2 ± 4.3	0.831	.478
	Within groups	5304.667	280	18.945			
	Total	5351.909	283				
Ferritin (ng/mL)	Between groups	552,361.370	3	184,120.457	444.9 ± 534.8	0.641	.589
	Within groups	80,678,113.700	281	287,110.725			
	Total	81,230,475.070	284				
Absolute neutrophil count (ANC) (cells/mL)	Between groups	3.770	3	1.257	3.6 ± 2.1	0.272	.846
	Within groups	1299.970	281	4.626			
	Total	1303.740	284				
Absolute lymphocyte count (cells/mL)	Between groups	71.192	3	23.731	1.4 ± 1.8	7.556	.000
	Within groups	882.478	281	3.140			
	Total	953.670	284				
Neutrophil to lymphocyte ratio (NLR)	Between groups	71.174	3	23.725	3.9 ± 5.1	0.899	.442
	Within groups	7418.801	281	26.401			
	Total	7489.976	284				
CD4 (cells/mm^3^)	Between groups	57,291.699	3	19,097.233	534.6 ± 268.0	0.264	.851
	Within groups	20,347,087.090	281	72,409.563			
	Total	20,404,378.790	284				
CD8 (cells/mm^3^)	Between groups	143,610.390	3	47,870.130	334.0 ± 209.7	1.089	.354
	Within groups	12,348,478.920	281	43,944.765			
	Total	12,492,089.310	284				
CD19 (cells/mm^3^)	Between groups	27,423.932	3	9141.311	164.4 ± 120.2	0.631	.596
	Within groups	4,073,875.844	281	14,497.779			
	Total	4,101,299.775	284				
NK (cells/μL)	Between groups	80,220.697	3	26,740.232	179.0 ± 172.6	0.897	.443
	Within groups	8,377,734.615	281	29,814.002			
	Total	8,457,955.312	284				
Fibrinogen (g/L)	Between groups	6.300	3	2.100	4.4 ± 1.4	1.037	.377
	Within groups	569.130	281	2.025			
	Total	575.430	284				
Haptoglobin (g/L)	Between groups	2.194	3	0.731	2.5 ± 1.2	0.498	.684
	Within groups	412.387	281	1.468			
	Total	414.581	284				
C3 (g/L)	Between groups	0.024	3	0.008	1.3 ± 0.2	0.150	.929
	Within groups	14.848	281	0.053			
	Total	14.871	284				
C4 (g/L)	Between groups	0.012	3	0.004	0.3 ± 0.1	0.619	.603
	Within groups	1.789	281	0.006			
	Total	1.801	284				
IgG (g/L)	Between groups	4.740	3	1.580	11.9 ± 2.3	0.299	.826
	Within groups	1483.679	281	5.280			
	Total	1488.419	284				
IgM (g/L)	Between groups	8.557	3	2.852	2.0 ± 3.7	0.207	.891
	Within groups	3868.046	281	13.765			
	Total	3876.603	284				
IgA (g/L)	Between groups	8.390	3	2.797	2.9 ± 1.0	2.893	.036
	Within groups	271.679	281	0.967			
	Total	280.069	284				
Beta 2 microglobulin (μg/mL)	Between groups	2.922	3	0.974	3.1 ± 1.0	0.972	.406
	Within groups	281.516	281	1.002			
	Total	284.438	284				
Zinc (μmol/L)	Between groups	0.837	3	0.279	10.7 ± 0.7	0.541	.654
	Within groups	144.293	280	0.515			
	Total	145.130	283				
LD (μmol/L)	Between groups	94,685.795	3	31,561.932	280.2 ± 148.3	1.441	.231
	Within groups	6,154,639.974	281	21,902.633			
	Total	6,249,325.768	284				
Iron level (μmol/L)	Between groups	57.694	3	19.231	8.5 ± 5.4	0.669	.572
	Within groups	8083.662	281	28.767			
	Total	8141.356	284				
Soluble transferrin receptor (mg/L)	Between groups	8.531	3	2.844	5.7 ± 3.1	0.299	.826
	Within groups	2671.767	281	9.508			
	Total	2680.298	284				
IgE (kU/L)	Between groups	1950.888	3	650.296	13.9 ± 34.8	0.534	.659
	Within groups	341,906.708	281	1216.750			
	Total	343,857.596	284				
ESR (mm/hr)	Between groups	2167.072	3	722.357	56.1 ± 38.2	0.493	.687
	Within groups	410,169.361	280	1464.891			
	Total	412,336.433	283				

The relationship between the results of radiological imaging of patients and their corresponding blood type is shown in Table 4. Although the analysis showed no statistically significant relationship between the blood group of the patient and the radiology outcomes, there were some observational differences. Out of all patients, 29.8% (n = 85) of cases had abnormalities in the radiological chest finding. Of those, 64.7% (n = 55) experienced bilateral lung abnormality. The most common X-ray findings among cases were infiltration and consolidation infiltrate. The blood types O and A were determined to have the highest abnormal chest X-ray outcome with a total percentage of O (31.3%) and A (30.9%).

**Table 4:** Radiologic findings variation between ABO blood groups.

Investigation	Variables	Categories	A	B	AB	O	*X* ^2^	*P*	Incidence
	Total	285 (100)	81 (28.4)	43 (15.1)	11 (3.9)	150 (52.6)			O>A>B>AB
X-ray imaging outcomes	Outcome	Normal	39 (48.1)	18 (41.9)	5 (45.5)	70 (46.7)	12.114	.207	O>A>B>AB
		Abnormal	25 (30.9)	11 (25.6)	2 (18.2)	47 (31.3)			O>A>B>AB
		Not done	17 (21.0)	14 (32.6)	4 (36.4)	33 (22.0)			O>A>B>AB
	Abnormality side	Right	5 (6.2)	0 (0.0)	0 (0.0)	10 (6.7)	8.426	.492	O>A
		Left	1 (1.2)	1 (2.3)	0 (0.0)	8 (5.3)			O>A=B
		Bilateral	16 (19.8)	10 (23.3)	1 (9.1)	28 (18.7)			O>A>B>AB
	Upper infiltrate	Yes	2 (2.5)	1 (2.3)	0 (0.0)	3 (2.0)	0.307	.959	O>A>B
		No	79 (97.5)	42 (97.7)	11 (100.0)	147 (98.0)			O>A>B>AB
	Middle infiltrate	Yes	7 (8.6)	0 (0.0)	0 (0.0)	9 (6.0)	4.656	.155	O>B
		No	74 (91.4)	43 (100.0)	11 (100.0)	141 (94.0)			O>A>B>AB
	Lower infiltrate	Yes	12 (14.8)	6 (14.0)	0 (0.0)	26 (17.3)	2.518	.472	O>A>B
		No	69 (85.2)	37 (86.0)	11 (100.0)	124 (82.7)			O>A>B>AB
	Not specified infiltrate	Yes	7 (8.6)	5 (11.6)	1 (9.1)	17 (11.3)	0.489	.921	O>A>B>AB
		No	74 (91.4)	38 (88.4)	10 (90.9)	133 (88.7)			O>A>B>AB
	Pleural effusion	Yes	5 (6.2)	3 (7.0)	0 (0.00)	11 (7.3)	0.931	.818	O>A>B
		No	76 (93.8)	40 (93.0)	11 (100.0)	139 (92.7)			O>A>B>AB
	Alveolar infiltrates	Yes	0 (0.0)	0 (0.0)	0 (0.0)	1 (0.7)	0.903	.825	O
		No	81 (100.0)	43 (100.0)	11 (100.0)	149 (99.3)			O>A>B>AB
	Interstitial infiltrates	Yes	3 (3.7)	3 (7.0)	0 (0.0)	3 (2.0)	3.146	.370	O>A>B
		No	78 (96.3)	40 (93.0)	11 (100.0)	147 (98.0)			O>A>B>AB
	Reticulonodular infiltrate	Yes	3 (3.7)	0 (0.0)	0 (0.0)	1 (0.7)	4.454	.216	O>A
		No	78 (96.3)	43 (100.0)	11 (100.0)	149 (99.3)			O>A>B>AB
	Congestion infiltrate	Yes	1 (1.2)	0 (0.0)	0 (0.0)	0 (0.0)	2.527	.470	A
		No	80 (98.8)	43 (100.0)	11 (100.0)	150 (100.0)			O>A>B>AB
	Atelectasis infiltrate	Yes	2 (2.5)	1 (97.7)	1 (9.1)	3 (98.0)	2.154	.541	O>A>B=AB
		No	79 (97.5)	42 (7.0)	10 (90.9)	147 (8.7)			O>A>B>AB
	Consolidation infiltrate	Yes	6 (7.4)	3 (93.0)	0 (0.0)	13 (8.7)	1.153	.764	O>A>B
		No	75 (92.6)	40 (4.7)	11 (100.0)	137 (91.3)			O>A>B>AB
CT scan imaging outcomes	Outcome	Normal	7 (8.6)	2 (11.6)	1 (9.1)	9 (6.0)	9.219	.417	O>A>B>AB
		Abnormal	6 (7.4)	5 (83.7)	1 (81.8)	13 (8.7)			O>A>B>AB
		Not Done	68 (84.0)	36 (11.6)	9 (9.1)	128 (85.3)			O>A>B>AB
	Abnormality side	Unilateral	1 (1.2)	0 (0.0)	0 (0.0)	3 (2.0)	2.570	.861	O>A
		Bilateral	5 (6.2)	5 (100.0)	1 (100.0)	10 (6.7)			O>A>B>AB
	Upper location	Yes	3 (3.7)	1 (2.3)	0 (0.0)	5 (3.3)	0.550	.908	O>A>B
		No	78 (96.3)	42 (97.7)	11 (100.0)	145 (96.7)			O>A>B>AB
	Middle location	Yes	0 (0.0)	0 (0.0)	0 (0.0)	0 (0.0)	-	-	-
		No	81 (100.0)	43 (100.0)	11 (100.0)	150 (100.0)			O>A>B>AB
	Lower location	Yes	1 (1.2)	3 (7.0)	1 (9.1)	9 (6.0)	3.529	.317	O>B>A=AB
		No	80 (98.8)	40 (93.0)	10 (90.9)	141 (94.0)			O>A>B>AB
	Not specified location	Yes	2 (2.5)	2 (4.7)	0 (0.0)	2 (1.3)	2.075	.557	O>A>B
		No	79 (97.5)	41 (95.3)	11 (100.0)	148 (98.7)			O>A>B>AB
	Ground glass opacity	Yes	6 (7.4)	3 (7.0)	1 (9.1)	10 (6.7)	4.700	.538	O>A>B>AB
		No	0 (0.0)	2 (4.7)	0 (0.0)	2 (1.3)			O>B
	Peribranchial thickening	Yes	1 (1.2)	0 (0.0)	0 (0.0)	0 (0.0)	4.368	.627	A
		No	4 (4.9)	5 (11.6)	1 (9.1)	11 (7.3)			O>A>B>AB
	Fibrosis	Yes	76 (93.8)	38 (88.4)	10 (90.9)	139 (92.7)	1.255	.740	O>A>B>AB
		No	5 (6.2)	5 (11.6)	1 (9.1)	11 (7.3)			O>A=B>AB
	Alveolar infiltrates	Yes	0 (0.0)	1 (2.3)	0 (0.0)	1 (0.7)	2.776	.836	O=B
		No	5 (6.2)	4 (9.3)	1 (9.1)	11 (7.3)			O>A>B>AB
	Pleural thickening	Yes	1 (1.2)	0 (0.0)	0 (0.0)	0 (0.0)	4.368	.627	A
		No	4 (4.9)	5 (11.6)	1 (9.1)	11 (7.3)			O>A>B>AB
	Pleural effusion	Yes	1 (1.2)	0 (0.0)	0 (0.0)	0 (0.0)	3.630	.727	A
		No	5 (6.2)	5 (11.6)	1 (9.1)	12 (8.0)			O>A=B>AB
	Tree in bud appearance	Yes	76 (93.8)	38 (88.4)	10 (90.9	139 (92.7)	1.255	.740	O>A>B>AB
		No	5 (6.2)	5 (11.6)	1 (9.1)	11 (7.3)			O>A=B>AB
	Consolidation	Yes	2 (2.5)	1 (2.3)	0 (0.0)	3 (2.0)	2.224	.898	O>A>B
		No	3 (3.7)	4 (9.3)	1 (9.1)	8 (5.3)			O>A>B>AB

There were no statistically significant relationships after comparing the findings of CT scans among patients with their blood groups, as seen in Table 4. There were abnormal CT chest findings in 8.8% (n = 55) of all cases. About 38.2% (n = 21) of the patients with abnormal chest CT scans had bilateral lung abnormalities. The most common imaging features seen in CT scan findings were fibrosis, bud appearance, and ground-glass opacity. The highest abnormal chest CT scan with a total percentage of O (8.7%) and A (7.4%) suggested a strong correlation between these two blood groups and the severity of COVID-19 infection (Table 4). However, the differences in both X-ray and CT scan findings were statistically nonsignificant among the ABO age group (Table 4).

Table 5 indicated that only 86 (30.3%) patients were admitted to an intensive care unit, and most of them were blood groups O (28.7% [n = 43]) and A (37.0% [n = 30]). Additionally, 54.6% (n = 47) of them received mechanical ventilation (MV), and their blood groups were O > A > B > AB. Only one patient (blood group A) received extracorporeal membrane oxygenation due to low oxygen levels. Additionally, results showed that two cases developed coma and their blood groups were B and O. Also, encephalitis and seizure were produced in four patients with blood groups O and A. Death was reported in blood groups O and A. Results presented that most complications were highly reported in group O then A, followed by group B and AB. This indicated that patients with blood group O have more difficulties than other groups; moreover, blood group AB has fewer complications. However, the differences in complications outcomes were statistically nonsignificant among the ABO age group (Table 5).

**Table 5:** Complications variation between ABO blood groups.

Variables	Categories	A	B	AB	O	*X* ^2^	*P*	Incidence
Total	285 (100)	81 (28.4)	43 (15.1)	11 (3.9)	150 (52.6)			O>A>B>AB
Admitted to an intensive care unit (ICU)	Yes	30 (37.0)	10 (23.3)	3 (27.3)	43 (28.7)	2.993	.393	O>A>B>AB
	No	51 (63.0)	33 (76.7)	8 (72.7)	107 (71.3)			O>A>B>AB
Patients receive mechanical ventilation (MV)/intubation	Yes	16 (19.8)	5 (11.6)	2 (18.2)	24 (16.0)	1.413	.702	O>A>B>AB
	No	65 (80.2)	38 (88.4)	9 (81.8)	126 (84.0)			O>A>B>AB
Patients receive extracorporeal membrane oxygenation (ECMO)	Yes	1 (1.2)	0 (0.0)	0 (0.0)	0 (0.0)	2.527	.470	A
	No	80 (98.8)	43 (100.0)	11 (100.0	150 (100.0)			O>A>B>AB
Coma	Yes	0 (0.0)	1 (2.3)	0 (0.0)	1 (0.7)	2.280	.516	O=B
	No	81 (100.0)	42 (97.7)	11 (100.0)	149 (99.3)			O>A>B>AB
Encephalitis	Yes	1 (1.2)	0 (0.0)	0 (0.0)	1 (0.7)	0.714	.870	O=A
	No	80 (98.8)	43 (100.0)	11 (100.0)	149 (99.3)			O>A>B>AB
Renal failure	Yes	5 (6.2)	1 (2.3)	2 (18.2)	8 (5.3)	4.226	.238	O>A>AB>B
	No	76 (93.8)	42 (97.7)	9 (81.8)	142 (94.7)			O>A>B>AB
Seizure	Yes	3 (3.7)	0 (0.0)	0 (0.0)	1 (0.7)	4.454	.216	A>O
	No	78 (96.3)	43 (100.0	11 (100.0)	149 (99.3)			O>A>B>AB
Sepsis	Yes	6 (7.4)	2 (4.7)	1 (9.1)	8 (5.3)	0.731	.866	O>A>B>AB
	No	75 (92.6)	41 (95.3)	10 (90.9)	142 (94.7)			O>A>B>AB
Symptoms resolved	Yes	62 (76.5)	32 (74.4)	9 (81.8)	116 (77.3)	0.319	.956	O>A>B>AB
	No	19 (23.5)	11 (25.6)	2 (18.2)	34 (22.7)			O>A>B>AB
Outcome at day fourteen after hospital admission	Cure	44 (54.3)	30 (69.8)	8 (72.7)	80 (53.3)	6.007	.422	O>A>B>AB
	Persistent disease	35 (43.2)	13 (30.2)	3 (27.3)	68 (45.3)			O>A>B>AB
	Death	2 (2.5)	0 (0.0)	0 (0.0)	2 (1.3)			O=A

## 4 Discussion

Since Karl Landsteiner discovered the ABO blood type system in 1901, researchers have been looking for a link between the ABO blood group system and numerous illnesses.^[17]^ The relationship between the ABO blood type and various illnesses, including bacterial and viral infections, has been thoroughly investigated.[^18^,^19^,^20^,^21^] The ABO blood group is now widely associated with various illnesses.^[22]^ Several recent investigations of COVID-19 infection in China and the United States have found a link between ABO blood types and infection severity and death. These investigations found that people with blood group A are more likely to get COVID-19 infection, whereas those with blood group O are less likely to contract COVID-19 infection. The severity of the illness may also be influenced by blood type. Individuals with blood type A had a lower risk of COVID-19 infection than those with other blood types.[^13^,^23^,^24^]

The current findings indicated that blood group O was the most prevalent blood group (52.6%) among the study participants, followed by A (28.4%), B (15.1%), and AB (3.9%). This was almost consistent with the distribution of blood groups among the Saudi Arabia community: blood group O (52%), A (26%), B (18%), and AB (4%).^[25]^ The results also showed that the upper respiratory tract infection and shortness of breath showed statistically significant differences between the ABO blood group (*P* = .014 and *P* = .009 retrospectively). Moreover, all symptoms were observed highly in blood group O followed by A then B except for pharyngeal exudate observed in blood group A. In addition, hemoptysis, jaundice, abnormal auscultation of the lung, and hepatomegaly were only reported in O blood groups. According to Wu Y et al 2020 and Komal et al 2021, COVID-19-infected people with blood types O and AB had a different clinical spectrum of signs and symptoms, with participants with the AB blood group having a slightly higher probability of fever and sore throat and a lower chance of losing their sense of taste and smell.[^19^,^26^]

The study indicated that among the studied hematological parameters, glucose (*P* = .004), absolute lymphocyte count (*P* = .001), and IgA (*P* = .036) showed statistically significant differences between the means of the ABO blood group. The findings of Kazancioglu et al 2020 revealed that eosinophils, lymphocytes, and platelet-to-lymphocyte ratio were the most critical factors in distinguishing COVID-19 patients from healthy controls.^[27]^ Sun et al found fewer eosinophils and lymphocytes, as well as a greater platelet-to-lymphocyte ratio, in COVID-19 patients compared to controls.^[28]^

Results showed that 29.8% (n = 85) and 8.8% (n = 55) of the study participants experienced abnormal chest findings using X-ray and CT scans, respectively. The most common results were infiltration and consolidation infiltrate. The blood types O and A were determined to have the highest abnormal chest outcome. In comparison to patients with other blood groups, Mansour et al 2021 discovered a significant association between patients with blood group A and a more severe pneumonic process in their non-contrast high-resolution CT chest with a comparatively higher severity score.^[29]^

Also, the findings indicated that only 86 (30.3%) patients were admitted to an intensive care unit, and the majority of them were blood groups O (28.7%, n = 43) and A (37.0%, n = 30). In the blood group analysis of the intensive care unit (ICU) COVID-19 patients, Yaylaci et al 2020 found that the distribution of patients’ blood group was O > B > A > AB (15.6%, 15.1%, 14.8%, 9.5%, respectively).^[30]^

Additionally, it is observed that 54.6% (n = 47) of admitted patients into the ICU received MV, and their blood groups were O > A > B > AB. Only one patient (blood group A) received extracorporeal membrane oxygenation due to low oxygen levels. Results showed that two cases developed coma and their blood groups were B and O. Also, encephalitis and seizure were produced in four patients with blood groups O and A. Death was reported in blood groups O and A. Results presented that most complications were highly reported in group O then A, followed by group B and AB. This indicated that patients with blood group O have more difficulties than other groups; moreover, blood group AB has fewer complications.

The multicenter retrospective research of Hoiland et al aimed at investigating if ABO blood types are linked to various severities of COVID-19 among ICU admitted patients and found that critically sick COVID-19 patients with blood group A or AB had a higher chance of needing MV and prolonged ICU length of stay compared with patients with blood groups O or B. ^[31]^ There were no significant variations in rates of ICU admissions, MV, vasopressors, acute renal failure, venous thromboembolism, and readmission rate between blood types A and O, according to Kumar et al.^[32]^ Non-O classes had a slightly higher infection prevalence, according to Zietz et al. When compared to type O, the risk of intubation was lower for type A and higher for types of AB and B. In contrast, the mortality risk was higher for type AB and lower for types A and B. Having a Rh-negative blood type protects an individual from all three consequences.^[33]^

The current study has several limitations that warrant consideration, such as primarily single-center, retrospective nature, small sample size, and no information about the strain of coronavirus. Future research will aim to address these limitations and unresolved questions.

## 5 Conclusion

The present study reported the susceptibility of the ABO blood group to COVID-19 infections. The clinical, laboratory, imaging findings, and symptoms variation were investigated. All symptoms were observed highly in blood group O except for pharyngeal exudate observed in blood group A. The findings in the current study may have a clinical recommendation that individuals with blood group O might need to particularly strengthen their immunity and personal protection to reduce the chances of getting COVID-19 infection. The overall symptoms trends of hospitalized COVID-19 patients belonging to different blood groups varied nonsignificantly. Larger scale replication research with comprehensive information should be encouraged to pursue and needed to verify the current outcomes.

## Acknowledgments

We would like to thank the King Faisal Specialist Hospital and Research Centre Riyadh and Jeddah, Saudi Arabia, for facilitating the conduction of this study. Also, the research team would like to acknowledge the deanship of scientific research for funding the project.

## Author contributions

**Administrative support:** Ahmed M. Kabrah, Arwa F. Flemban, Fayez S. Bahwerth, Hamza M. Assaggaf, Hebah M. Kabrah.

**Collection and assembly of data:** Sarah A. Al-Shareef, Wesam H. Alsabban, Anmar A. Khan, Fayez S. Bahwerth, Hebah M. Kabrah.

**Conception and design:** Saeed M. Kabrah, Samer S. Abuzerr, Mohammed A. Boghdadi.

**Conceptualization:** Saeed M. Kabrah, Samer S. Abuzerr, Mohammed A. Boghdadi, Ahmed M. Kabrah, Arwa F. Flemban, Fayez S. Bahwerth, Sarah A. Al-Shareef, Wesam H. Alsabban, Akhmed M. Aslam.

**Data analysis and interpretation:** Hessah A. Alzhrani, Layal K. Jambi, Radi T. Alsafi, Ahmad O. Babalghith, Akhmed M. Aslam.

**Data curation:** Fayez S. Bahwerth, Hessah A. Alzhrani, Hebah M. Kabrah, Ahmad O. Babalghith.

**Final approval of manuscript:** All authors.

**Formal analysis:** Eisa A. Alanazi, Radi T. Alsafi.

**Investigation:** Radi T. Alsafi.

**Manuscript writing:** All authors.

**Methodology:** Sarah A. Al-Shareef.

**Provision of study materials or patients:** Eisa A. Alanazi, Abdullah A. Alhifany, Amr J. Halawani.

**Software:** Hessah A. Alzhrani, Ahmad O. Babalghith, Amr J. Halawani.

**Supervision:** Saeed M. Kabrah, Samer Saleem S. Abuzerr.

**Validation:** Saeed M. Kabrah, Samer Saleem S. Abuzerr, Mohammed A. Boghdadi, Anmar A. Khan, Layal K. Jambi.

**Writing – original draft:** Anmar A. Khan, Layal K. Jambi, Ahmad O. Babalghith.

**Writing – review & editing:** Hamza M. Assaggaf, Abdullah A. Alhifany, Anmar A. Khan, Amr J. Halawani, Ahmad O Babalghith.
